# Factors influencing childhood obesity – the establishment of a population-wide monitoring system in Germany

**DOI:** 10.17886/RKI-GBE-2017-047

**Published:** 2017-06-14

**Authors:** Gianni Varnaccia, Johannes Zeiher, Cornelia Lange, Susanne Jordan

**Affiliations:** Robert Koch Institute, Department for Epidemiology and Health Monitoring, Berlin, Germany

**Keywords:** OBESITY, INDICATORS, HEALTH MONITORING, CHILDREN, PREVENTION

## Abstract

Obesity poses a danger to childhood health and can continue to have a negative impact on health into adulthood. Currently, about 15% of children and adolescents in Germany are overweight or obese. Moreover, significant data on the multifactorial causes of childhood obesity that is systematically recorded, regularly updated and obtainable at the nationwide level are not yet available in Germany. As such, the Robert Koch Institute is establishing a population-wide system to monitor the factors that are relevant to childhood obesity (AdiMon). AdiMon will be available by the end of 2017. This paper outlines the methodological approach that is being used to establish AdiMon and describes the current results of the project (the development of an initial set of core indicators).

The project began by undertaking a systematic literature review aimed at piecing together the latest knowledge on factors that influence childhood obesity. The factors that were identified were then sorted according to relevance, and appropriate indicators were selected. This was followed up by research into data sources that – as far as possible – provide significant data that are regularly collected but that also provide for regional differentiation. Work is currently underway to analyse these indicators and data sources. Once this work has been completed, the indicator set will be finalised and the results published on the internet.

Population-wide monitoring of factors relevant to childhood obesity takes the following types of indicators into account: behavioural factors (such as physical activity), biological factors (such as genetic predisposition), prenatal and early-childhood factors (such as breastfeeding), psychosocial factors (such as parents’ health consciousness), environmental factors (such as playgrounds in the local area), contextual factors (such as a migrant background) and prevention measures as well as measures to promote health (such as expenditure by statutory health insurers). The population-wide monitoring uses the following data sources: epidemiological studies, social sciences surveys, official statistics and geo-information systems, as well as routine, economic and media data.

This paper demonstrates that population-wide monitoring can provide significant information about the distribution and causes of obesity in childhood, and thus enable the need for action to be recognised at an early stage, initial approaches for preventive measures to be identified and developments to be tracked over time.

## 1. Introduction

Obesity is one of the greatest public health policy challenges of the 21st century [1]. In fact, it can even endanger health in childhood and can continue to have a negative impact on health into adulthood [2, 3]. As early as 2003, Germany launched its own public health initiative ‘growing up healthy’, which contributes to the prevention of childhood obesity by encouraging exercise and a healthy diet [4]. In 2014, the European Commission launched the EU Action Plan on Childhood Obesity [5]. Similarly, a group of organisations, including the World Health Organization (WHO) and the European Commission, are calling for the implementation of comprehensive monitoring measures to counter the distribution of obesity in childhood [5, 6].

**Info box 1: Definition Monitoring**

Monitoring is the continuous or periodic systematic collection of data for the surveillance of processes and results [9].

About 15% of children and adolescents in Germany are classified as overweight or obese [7]. Nevertheless, systematically recorded, regularly updated, significant datasets on the multifactorial causes of childhood obesity are still not available at the national level in Germany. As such, the Robert Koch Institute is establishing a population-wide monitoring system of factors that influence childhood obesity (AdiMon; with focus from 0 to 6 years of age). The system is being funded by the Federal Ministry of Health and it will be available by the end of 2017. AdiMon focuses on 0- to 6-year-olds because this stage of life is crucial in obesity prevention [8]. On the one hand, some influencing factors (such as breastfeeding) are only relevant during this stage of life; on the other, additional factors (such as dietary behaviour) are considerably shaped during this period. In addition, as there is a marked increase in the prevalence of obesity among children of school age, it is important to assess the causes of childhood obesity in children below this age [7]. The indicator system developed for population-wide monitoring is intended to lead to a pool of scientifically supported information about the causes and distribution of childhood obesity, and thus the opportunity to recognise the need for action at an early stage, identify initial approaches to preventive measures and track developments over time. This paper describes the methodological approach used to develop AdiMon and explains the current status of the project (the development of a set of core indicators).

## 2. Methods

**Figure 1:**
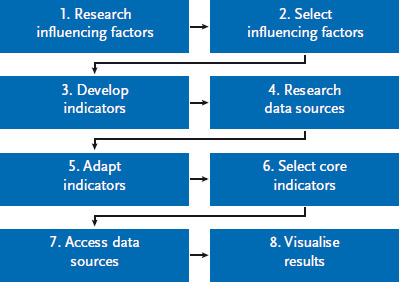
The steps used to develop a population-wide monitoring system of factors that influence childhood obesity Source: own diagram

The following describes the methodological approach used to establish the population-wide monitoring of factors that influence childhood obesity. The approach can be divided into eight steps (Figure 1).

**Info box 2: Definition Indicator**

An indicator is an empirically measurable, observable or analysable dimension.

Indicators are useful in assessing (usually complex) situations that cannot be directly measured or evaluated [9].

### 2.1 Research into influencing factors

A systematic literature review was carried out in order to bring together current knowledge about factors that influence childhood obesity. Detailed information on the literature review and the subsequent selection of influencing factors can be found in Zeiher et al. [10]. Both risk-related and protective factors were considered so as to provide the most comprehensive overview possible of the multifactorial causes of childhood obesity. Factors were considered if they are causally associated with obesity in childhood, or where they are linked to childhood obesity but the causal relationship has yet to be sufficiently explored.

### 2.2 Selection of influencing factors

Four criteria of exclusion were developed so that influencing factors relevant to monitoring could be selected. Factors were not taken into account if they only affected small parts of the population (such as genetic disorders), if they had little bearing on the age group in question (0 to 6 years – such as medicine intake), were not important for Germany (such as climate), or if a majority of the studies included in the systematic literature reviews had been unable to demonstrate any relationship between the factor in question and the development of childhood obesity (such as milk consumption).

### 2.3 Developing the indicators

The selected influencing factors were supplemented by ‘ideal type’ indicators. Ideal type indicators are formulated independently of a particular data source and describe the corresponding influencing factor in the best possible manner. Work on formulating the indicators also took into account the ZWERG guidelines (central importance, economic efficiency, simplicity, timeliness, accuracy) [11]. These guidelines stipulate that indicators should provide significant information that reflects the aim of the work being undertaken, be generally understandable, plausible, collectable using a reasonable amount of resources, available at an appropriate time and constitute reliable benchmarks.

### 2.4 Research into the data sources

A search was conducted for suitable data sources that could equip the indicators with the necessary data. To this end, a range of areas were investigated. First, the usual sources of data used in health reporting were examined. These include epidemiological studies (such as the ‘German Health Interview and Examination Survey for Children and Adolescents’ – the KiGGS study [12]), social scientific surveys (such as ‘Growing up Healthy in Germany: Everyday life AID:A Study’ [13]), official statistics (such as microcensuses [14]) as well as routine data (such as from the Prevention Report published by statutory health insurers [15]). Furthermore, a review of scientific databases (Scopus, PubMed and Google Scholar) was conducted to find publications with references to relevant data sources. Grey literature was identified using the Google search engine, and geo-information systems (such as OpenStreetMap) were analysed for relevant content. If several suitable data sources were available for the same indicator, the source that provided the most relevant data - that was regularly collected and which permitted regional differentiations to be made - was chosen.

**Info box 3: Selecting a core indicator**

The consumption of sweetened beverages soft drinks is one of the core indicators related to behavioural factors (Table 1). This indicator was selected because it complied best with the selection criteria and was viewed as particularly relevant by external experts at a workshop. The indicator is based on convincing evidence [16] and the data source (the KiGGS study) provides population-based, significant and regularly collected data. In addition, it is easily understandable (for example, compared to daily energy intake), significant (as an indicator of unhealthy dietary habits), dynamic (it clearly shows changes in consumption habits) and is found widely among the population.

### 2.5 Adaptation of the indicators

If a data source was available for an influencing factor, but the ideal-type indicator could not be used, the indicator was adapted accordingly. For example, an age restriction was placed on an indicator if a data source provided no information about the entire agegroup (0-6 years).

### 2.6 Selection of the core indicators

In order to highlight indicators that are particularly important and to enable quick access to the indicator system, a set of core indicators was selected for the population-wide monitoring system. Core indicators were selected according to the following criteria: strong evidence of a relation to obesity; the availability of significant data that was collected regularly in a population-wide manner and that provided for regional differentiation; the factor demonstrated a high distribution among the population in question, and had a high level of significance for its particular field of influence; as well as clarity and a timely response to changes. At a workshop with external experts, these criteria were used to develop a selection of core indicators that could serve as a basis for the population-wide monitoring.

### 2.7 Access the data sources

Work on extracting the data from the data sources will have been completed by August 2017. This work represents part of the penultimate phase of the project. Currently, relevant data is still being extracted and evaluated from freely available data sources, and requests for supplementary information from the data holders for specific indicators are being sent out (February 2017).

### 2.8 Visualisation of the results

The indicator system is due to be published online at the end of 2017. AdiMon will be made freely accessible via the Robert Koch Institute’s main website (www.rki.de/adimon). The website will provide users with comprehensive information about the distribution and causes of childhood obesity, enable the need for action to be recognised at an early stage, as well as help identify initial approaches to preventive measures and trends over time. The website is being designed to reflect the needs of its users to ensure that the results can be visualised in a user-friendly manner. The content-related, formal and graphical requirements of websites of this kind were discussed at a workshop with external experts from the scientific community and from municipal and regional health reporting. Wherever possible, the website will provide a gender-specific representation and description of the indicators. In addition, links to the data sources are to be made available in order to provide access to the latest data. After the project has ended, indicators that are based on periodic surveys will be kept up-to-date.

## 3. Results

**Figure 2:**
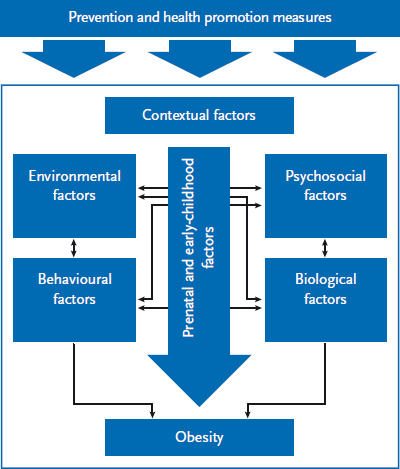
Simplified cause-and-effect model of childhood obesity Source: own diagram

A systematic literature review led to the identification of more than 60 influencing factors that are relevant to the development of childhood obesity [10]. These factors were used to construct a simplified cause-and-effect model of childhood obesity (Figure 2). In accordance with this model, obesity is caused by behavioural factors (such as physical activity) and biological factors (including genetic predisposition). Prenatal factors (such as maternal weight gain) and early-childhood factors (such as breastfeeding) also influence childhood obesity. Furthermore, psychosocial factors (such as parents’ health consciousness), environmental factors (such as playgrounds in the local area) and contextual factors (including a migrant background) also have an impact. Finally, measures in prevention and health promotion are also relevant for the distribution of obesity in childhood.

More than 100 indicators were developed for population-based monitoring that provide information about numerous influencing factors and the distribution of childhood obesity. Of these, 26 core indicators were selected and are presented below following the domains of the simplified cause-and-effect model.

### 3.1 Behavioural factors

A balanced diet [16], physical activity [17], and adequate sleep [18] help prevent the development of childhood obesity. Core indicators in terms of behavioural factors are the ‘proportion of children who drink sweetened refreshments daily’, the ‘proportion of children who eat fruit and vegetables daily’, the ‘proportion of children who meet the WHO’s recommendations on physical activity levels’ and ‘the number of hours children sleep per day’ from the KiGGS study [12], as well as the ‘the daily amount of time spent by children watching television’, which is collated by the Arbeitsgemeinschaft Fernsehforschung (AGF) [19] (Table 1).

**Info box 4: Definition Childhood obesity**

Obesity in childhood is often determined using the Body Mass Index (BMI). The BMI is calculated using a child’s height and weight (BMI=kg/m^2^) which is then compared to age- and gender-specific reference values. If a child’s BMI is above this reference value, they are regarded as obese. In Germany the Kromeyer-Hauschild reference values are generally used (with obesity defined as a BMI higher than the 97th percentile) [50].

### 3.2 Biological factors

Genetic factors (such as genetic predisposition [20]) and hormonal factors (such as leptin resistance [16]), microbiological factors (including intestinal flora [21]) and certain illnesses (such as those caused by Adenoviruses [21]) can encourage the development of obesity in childhood. Due to the lack of suitable data sources, no indicators could be formulated that appropriately described biological factors. Nevertheless, the indicator ‘proportion of parents who are overweight or obese’, which stems from the microcensus [14] and is located within the field of environmental factors to describe the family environment, provides information on genetic predispositions (Table 1).

### 3.3 Prenatal and early childhood factors

During the crucial prenatal and early-childhood phase, a normal increase in the weight of the mother during pregnancy [22], and breastfeeding [23] help prevent childhood obesity. The ‘proportion of mothers who had a high weight gain during pregnancy (> 30%)’ from evaluations conducted by the Institute for Quality Assurance and Transparency in Healthcare (IQTIG) [24] and the ‘proportion of children who were ever breastfed’, taken from the KiGGS study [12] (Table 1), were therefore selected as provisional core indicators of prenatal and early childhood influencing factors.

### 3.4 Psychosocial factors

Psychosocial factors that encourage the development of obesity in children include specific personality traits (such as low self-regulation [25]), emotional regulation mechanisms (such as reactions to stress [26]) and a lack of protective factors (such as insufficient social resources [27]). In addition, parental psychosocial factors are also associated with the development of childhood obesity. These include a lack of health literacy [28], psychological disorders (such as depression [29]) and parental perceptions of a child’s body weight [30]. Due to insufficient or unsuitable data sources, only a few psychosocial influencing factors could be mapped properly with indicators. Core indicators in terms of psychosocial factors are the ‘proportion of parents who place a high or very high level of importance on their personal health’ and the ‘proportion of parents who have been diagnosed with depression or depressive moods during the last 12 months’ from the ‘German Health Update’ (GEDA) [31], as well as the ‘proportion of parents who do not judge their obese child to be overweight’ from the KiGGS study [12] (Table 1).

### 3.5 Environmental factors

Access to a balanced diet [32, 33], opportunities for age-appropriate exercise [27], as well as health-promoting conditions in nurseries [34] and the family environment [35, 36] help to counteract the development of obesity in childhood. In contrast, environmental factors such as advertising for certain foods [37] can have a negative effect on childhood obesity. The following were selected as core indicators of environmental factors: the ‘proportion of recreational areas in urban areas’ from area statistics [38], the ‘number of playgrounds per 10,000 inhabitants’ and the ‘number of fast-food restaurants per 10,000 inhabitants’ from OpenStreetMap [39]. In addition, the ‘consumer price index for fruit, vegetables and confectionery’ and the ‘consumer price index for sports and recreational services’ were sourced from calculations made by the Federal Statistical Office (Table 1) [40]. The ‘proportion of nurseries whose catering adheres to external quality standards’, drawn from the Catering in Nurseries study (VeKiTa) [41], was chosen as a core indicator as it provides insights into health-promoting conditions in nurseries. A child’s family environment is described by the following core indicators: the ‘proportion of parents who eat fruit and vegetables daily’, the ‘proportion of parents who take part in sport’ both from the GEDA study [31], the ‘proportion of parents who are overweight or obese’, from the microcensus [14], and the ‘proportion of parents who go to the playground with their child several times a week’ from the AID:A study [13].

### 3.6 Contextual factors

In addition to the influencing factors mentioned so far, population-wide monitoring also takes into account contextual factors that are related to childhood obesity. These factors include socio-demographic [42] and cultural factors [43]. ‘Parental educational level’ and ‘proportion of children with a migrant background’ from the microcensus [14], as well as the ‘proportion of children who live in households that receive benefits in accordance with SGB II’ from the social security statistics provided by the German Federal Employment Agency [44] were selected as provisional core indicators in this case (Table 1).

### 3.7 Measures in prevention and health promotion

Population-wide monitoring needs to supply information about prevention and health promotion measures that can be used to counteract childhood obesity [45]. These include policy- [46] and setting-related measures [34, 47]. The indicators ‘implemented policy measures’ (such as drawing up appropriate statutory provisions at the national level to implement the EU School Fruit and Vegetables Scheme), from the World Cancer Research Fund International’s NOURISHING framework [48], as well as ‘expenditure by statutory health insurers on prevention measures in nurseries’ from the Prevention Report compiled by statutory health insurers [15] were selected as preliminary indicators (Table 1).

### 3.8 Obesity

The distribution of childhood obesity is described by the core indicator ‘proportion of 3- to 6-year-old children who are overweight or obese’ (Table 1) from the KiGGS study [12]. In addition, a core indicator based on the physical examinations that are undertaken when children begin school is planned so as to provide small-scale findings about the distribution of obesity at the end of the preschool phase. However, it will only be possible to implement this once it has become clear that the relevant data can be used promptly and regularly for population-wide monitoring.

## 4. Discussion

The population-wide monitoring of factors influencing childhood obesity comprises more than 100 indicators, 26 of which constitute core indicators at the present time. In order to provide significant information about the distribution and causes of childhood obesity, data is being extrapolated from sources covering several disciplines. Similar forms of monitoring in the fields of nutrition and exercise have been conducted in countries such as Switzerland [51]. In these cases, established indicators from various institutions were combined and in some situations new indicators were developed. For several years now, this has provided Switzerland with comprehensive information about the nutrition and physical activity situation of its entire population, and the data it has resulted in are now being used to develop preventive measures.

Population-wide monitoring of factors relevant to childhood obesity faces a limitation due to varying evidence levels behind the considered influencing factors [10]. Whereas numerous high-quality studies are available for certain influencing factors (such as breastfeeding), other influencing factors have only been investigated to a limited extent (for example, intestinal flora). Furthermore, there are also large differences in the availability of suitable data sources. As data sources were not available for some indicators, AdiMon cannot adequately describe certain areas that are influenced by particular factors (such as biological factors). A further limitation is caused by differences in the quality of data sources that are available. It was impossible to find data sources that were based on valid measurement instruments and large samples for all indicators. In addition, some data are not collected continuously or after short intervals, and others do not provide for small-scale comparisons. For example, OpenStreetMap is a data source that provides population-wide information on environmental influencing factors, but its validity is dependent on the number and activity of its members who provide user-generated content; thus, validity varies regionally. Therefore, in the course of further analysis of the data sources and the ongoing development of the indicator system, it is possible that some of the indicators presented in this paper will not be included in the final indicator set.

## 5. Conclusion and outlook

Despite these limitations, AdiMon will provide important information about the causes and distribution of obesity in childhood. Therefore, it will enable the need for action to be recognised at an early stage, initial approaches for preventive measures to be identified and developments to be tracked over time.

AdiMon is to be published on the Robert Koch Institute’s website by the end of 2017. For this purpose, supplementary information for individual indicators has been requested from data holders. In addition, a customised website structure is being designed so that the results of monitoring can be represented visually in a user-friendly manner. The freely accessible monitoring system is intended to provide current data and therefore contribute towards the development of further measures aimed at preventing childhood obesity. AdiMon is also intended as a means of mapping long-term population-wide developments within childhood obesity and its influencing factors. Finally, the health monitoring conducted by the Robert Koch Institute provides an important data basis that has been linked to high-quality and innovative data sources as part of the AdiMon project, and this will enable comprehensive and substantial monitoring of the factors influencing childhood obesity to be undertaken.

## Key statements

- AdiMon is a population-wide monitoring system that examines factors that influence childhood obesity.
- AdiMon provides information about more than 60 factors that influence childhood obesity.
- AdiMon comprises over 100 indicators and, at the current time, 26 core indicators.
- Regional comparisons are possible, even partly at the district level.
- AdiMon will be made freely available via the internet at the end of 2017.

## Figures and Tables

**Table 1:** Core indicators in population-wide monitoring of factors influencing childhood obesity (as of February 2017) Source: own diagram

Model area	Core indicator	Data Source
Obesity	Proportion of 3- to 6-year-old children who are overweight or obese	KiGGS study [12]
Behavioural factors	Proportion of 3- to 6-year-old children who drink sweetened refreshments daily	KiGGS study [12]
	Proportion of 3- to 6-year-old children who eat fruit and vegetables daily	KiGGS study [12]
	Proportion of 3- to 6-year-old children who meet the WHO’s recommendations on physical activity levels	KiGGS study [12]
	The daily amount of time spent by 3- to 5-year-old children watching television	AGF evaluation [19]
	The number of hours 0- to 6-year-old children sleep per day	KiGGS study [12]
Prenatal and early childhood factors	Proportion of mothers who had a high weight gain during pregnancy (> 30%)	IQTIG evaluation [24]
	Proportion of 0- to 6-year-old children who were ever breastfed	KiGGS study [12]
Psychosocial factors	Proportion of parents of 0- to 6-year-old children who place a high or very high level of importance on their personal health	GEDA study [31]
	Proportion of parents of 0- to 6-year-old children who have been diagnosed with depression or depressive moods during the last 12 months	GEDA study [31]
	Proportion of parents of 3- to 6-year-old children who do not judge their obese child to be overweight	KiGGS study [12]
Environmental factors	Number of fast-food restaurants per 10,000 inhabitants	OpenStreetMap [39]
	Consumer price index for fruit, vegetables and confectionery	Consumer price index [40]
	Proportion of recreational areas in urban areas	Area statistics [38]
	Number of playgrounds per 10,000 inhabitants	OpenStreetMap [39]
	Consumer price index for sports and recreational services	Consumer price index [40]
	Proportion of children’s nurseries whose catering adheres to external quality standards	VeKiTa study [41]
	Proportion of parents who eat fruit and vegetables daily	GEDA study [31]
	Proportion of parents who take part in sports	GEDA study [31]
	Proportion of parents who are overweight or obese	Microcensus [14]
	Proportion of parents who go to the playground with their child several times a week	AID:A study [13]
Contextual factors	Educational level of parents of 0- to 5-year-old children	Microcensus [14]
	Proportion of 0- to 6-year-old children who live in households that receive benefits in accordance with SGB II	Social security statistics [44]
	Proportion of 0- to 5-year old children with a migrant background	Microcensus [14]
Measures of prevention and health promotion	Implemented policy measures	NOURISHING database [48]
	Expenditure by statutory health insurers on preventive measures in nurseries	Prevention report [15]

KiGGS=German Health Interview and Examination Survey for Children and Adolescents; AGF=Arbeitsgemeinschaft Fernsehforschung; IQTIG=Institute for Quality Assurance and Transparency in Healthcare; GEDA=German Health Update; WHO=World Health Organization; VeKiTa=Catering in Nurseries; AID:A=Growing up Healthy in Germany: Everyday life; SGB=German Social Code
